# CircRNA ciRS-7: a Novel Oncogene in Multiple Cancers

**DOI:** 10.7150/ijbs.54292

**Published:** 2021-01-01

**Authors:** Junwen Chen, Jun Yang, Xiang Fei, Xia Wang, Kefeng Wang

**Affiliations:** 1 Department of Urology, Shengjing Hospital of China Medical University, Shenyang 110004, China.; 2 Department of Gastroenterology, Shengjing Hospital of China Medical University, Shenyang 110004, China.

**Keywords:** ciRS-7, oncogene, cancers

## Abstract

circular RNA ciRS-7 (ciRS-7) is a type of endogenous circular RNA (circRNA) with a closed circular structure. Since Hansen first demonstrated that ciRS-7 could serve as a microRNA sponge in 2013, researchers have paid increased attention to this circRNA. ciRS-7 plays a crucial role in regulating RNA transcription, downstream gene expression, and protein production. Moreover, ciRS-7 acts as an oncogene and promotes tumor progression through competitively inhibiting miR-7 in various types of cancers. ciRS-7 has been identified to be closely associated with breast cancer, nasopharyngeal carcinoma, lung cancer, hepatocellular carcinoma, cervical cancer, osteosarcoma, melanoma, colorectal cancer, esophageal squamous cell carcinoma, gastric cancer, pancreatic cancer, laryngeal squamous cell carcinoma, and cholangiocarcinoma. In this review, we summarize the biological characteristics, molecular mechanisms, and future challenges of ciRS-7 in multiple tumors.

## Introduction

Cancer represents a significant challenge for humans and is the second leading cause of death in the United States 1. Cancer mortality has gradually decreased since 1991 due to the substantial efforts that have been made in the fight against cancer 1. Although several diagnostic and therapeutic achievements have been made to combat cancer, our understanding of anti-tumor mechanisms remains incomplete.

Circular RNAs (circRNAs) were identified in both viruses and viroids in the late 1970s 2. With the development of high-throughput sequencing technology, circRNAs were demonstrated to be stable in several tissues and human fluids 3. Over the past few years, circRNAs have been found to play a vital role in tumorigenesis and cancer progression 4, 5. For example, circular RNA circZNF566 could promote hepatocellular carcinoma cell migration, invasion, and proliferation through sponging miR-4738-3p 4. In addition, AR-circHIAT1/miR-195-5p/29a-3p/29c-3p/CDC42 signaling could promote clear cell renal cell carcinoma cell migration and invasion 5. Therefore, the mechanism of circRNAs should be summarized and further studied.

circRNA ciRS-7 (ciRS-7) was first identified by Hansen in 2011, which was originated from cerebellar degeneration-related protein 1 antisense transcript (CDR1AS) 6. He also verified that ciRS-7 was absent with 3' poly-A tail and 5' cap, which confirmed the circular structure 6. Therefore, ciRS-7 is also named CDR1as or CDR1NAT and functioned as a circRNA in many human diseases. The host gene of ciRS-7 is located on chromosome Xq27.1, which contains about 1500 nt lengths and more than 70 seed regions for miRNAs, especially for miR-7 7. ciRS-7 is generated through back splicing between flanking splice regions and spliced into linear transcripts like other circRNAs 8. The biogenesis of ciRS-7 is promoted by short interspersed nuclear elements, which lies in an inverted orientation of the downstream and upstream of ciRS-7 exon. With the development of study, we find that ciRS-7 is considered to be a competing endogenous RNA (ceRNA) due to its intimate relationship with miR-7 7, 9. In recent years, ciRS-7 was found to function as an oncogene and play a vital role in various types of cancers, including lung cancer, hepatocellular carcinoma (HCC), cervical cancer (CC), osteosarcoma (OS), melanoma, colorectal cancer (CRC), breast cancer (BC), esophageal squamous cell carcinoma (ESCC), nasopharyngeal carcinoma (NPC), gastric cancer (GC), pancreatic ductal adenocarcinoma (PDAC), laryngeal squamous cell carcinoma (LSCC), and cholangiocarcinoma 10-22. In this review, we will summarize the research status regarding the mechanism and clinical significance of ciRS-7 in the onset and progression of cancer.

## Functions of circRNAs

### Function as a biomarker

Increased evidence has shown that circRNAs could be used as biomarkers for the diagnosis and prognosis of several diseases 23, 24, particularly tumors 25, 26. A study by Jiang et al. 27 found that hsa_circ_0004904 and hsa_circ_0001855 may represent a promising prognostic biomarker of preeclampsia. Moreover, Ouyang et al. 28 reported that the upregulation of circRNA_002453 was associated with the severity of lupus nephritis, which could represent a potential diagnostic biomarker. In addition, circRNA_0000285 may be a novel biomarker for NPC 29 and hsa_circ_0003159 could potentially serve as a diagnostic marker of GC 30. Shao et al. 31 found that hsa_circ_0000705 had a strong sensitivity and specificity for GC. Moreover, recent studies have shown that exosomal circular RNA could be used to distinguish cancers from that of normal tissues. exo-hsa_circRNA_0056616 was also found to serve as a potential biomarker in lung adenocarcinoma with lymph node metastasis 32. Additionally, circ-PDE8A was reported to be associated with PDAC 33.

### Function as a microRNA (miRNA) sponge

circRNAs have miRNA binding sites on their sequences, which represent the basis of miRNA sponges 7. This property suggests that circRNAs can inhibit the activity of mature miRNAs and increase the level of endogenous targets and suppress miRNA to regulate downstream gene expression 34. There are multiple circRNAs that can bind to miRNA and have a different functional role in human diseases. For instance, circMMP9 could sponge miR-124 to promote glioblastoma multiforme cell proliferation, migration, and invasion 35. circWDR77 sponges miR-124 to regulate vascular smooth muscle cell proliferation and migration in the cardiovascular system 36. One circRNA is capable of targeting several miRNAs via different binding sites and performing opposite functions. For instance, circ-ITCH could promote the migration, invasion, and growth of OS cells through the miR-7/EGFR pathway 37. Moreover, circ-ITCH can also inhibit cellular prolifaration, migration, invasion, and metastasis through sponging miR-17/miR-224/p21/PTEN signaling in bladder cancer 38.

### Protein binding

In addition to miRNA binding, some circRNAs have been found to bind to RNA binding proteins (RBPs) through specific binding sites 39-43. The study by Dai et al. 39 found that the RBP trinucleotide repeat-containing 6A could bind to the flanked intron region of circ0006916 and regulate its biogenesis. Chen et al. 40 revealed that circAGO2 could interact with the human antigen R protein (HuR) to perform HuR-repressed AGO2-miRNA complexes functions. It was later discovered that circ-HuR could bind to CCHC-type zinc finger nucleic acid binding protein to reduce HuR expression and suppress tumor progression in GC 41. In addition, circRNAs can bind to proteins to form functional complexes. For example, circFOXK2 could interact with the Y Box binding protein 1 and heterogeneous nuclear ribonucleoprotein K to form a complex, which could enhance NUF2 and PDXK expression in PDAC 42. Another study showed that circSamd4 could form a complex with PUR proteins to enhance myogenesis through the suppression of myosin heavy chain transcription 43.

### Protein translation

circRNAs were formerly considered to be incapable of protein translation. Recently, researchers have revealed that some circRNAs with open reading frames (ORFs) could be translated to produce proteins 44. The study by Abe et al. 45 reported that circRNAs with an ORF were translated into proteins in rabbit reticulocyte lysates. Pamudurti et al. 46 showed that circRNAs could be translated to produce proteins in flies. Moreover, Legnini et al. 47 reported that circ-ZNF609 could produce proteins in a splicing-dependent and cap-independent manner. circ-AKT3 encoded a novel protein (AKT3-174aa), which decreased the proliferation and radiation resistance of glioblastoma cells 48. Moreover, hsa-circ-0000423 could encode a functional protein (circPPP1R12A-73aa) to promote the proliferation, migration, and invasion of colon cancer cells 49. circFNDC3B was found to encode a novel protein (circFNDC3B-218aa) that could induce an inhibitory effect on colon cancer cells 50.

## Pathogenic mechanism of ciRS-7 in cancers

### Maintaining proliferative signals

Cell proliferation is one of the most important characteristics of cell life and the basis of biological reproduction, maintaining the relative balance of the number of individual cells and the normal functions of the body. Cell proliferation can help wound healing, tissue regeneration and pathological tissue repair. The regulation of proliferation is rigorous, entire monitoring, and involved in multiple layers. The production and release of growth-promoting factors during the whole cell division cycle should be well controlled to insure tissue homeostasis. This regulating control system is dysfunctional in cancer cells.

ciRS-7 is identified to maintain cell proliferation by several mechanisms in many kinds of cancers. A knockdown of ciRS-7 can inhibit cell proliferation through inducing G0/G1 arrest or increasing G1/S transition in lung cancer 10, OS 13, and CRC 15. In addition, ciRS-7 can also promote cell growth through sponging multiple miRNAs (e.g., miR-1270 51, miR-7 17, 18, 21, miR-876-5p 52, miR-942 42 ) in tumors.

### Avoiding cellular death

Programmed cellular death is a kind of active and orderly way of death, which is determined by genes. Specifically, it refers to suicidal protective measures initiated by gene regulation when cells are stimulated by internal and external environmental factors. In this way, humans remove non-essential cells or cells that are about to undergo specialization, including the induction of activation and genetic programming by a number of molecular mechanisms. When programmed cellular death occurred, apoptotic cells dispersed in normal tissue cells without inflammatory response or scar. The dead cellular debris is quickly removed by macrophages or neighboring cells without affecting the normal functions of other cells. There are two types of genes that control programmed cellular death. One type of gene inhibits cellular death, and the other promotes or initiates cellular death. These genes interact with each other to control normal cellular death. Cancer cells display increased tolerance to both genomic and environmental stresses, leading to resistance to tumor apoptosis.

The manipulation of ciRS-7 (upregulation or downregulation) can influence the cell apoptosis through EGFR or other signalings in lung cancer 10, 53, 54, OS 13, and CRC 55. Additionally, ciRS-7 is also reported to inhibit apoptosis and then decrease the sensitivity of tumor cells to drug resistance in multiple tumors 56-60.

### Inducing invasion and metastasis

Tumor invasion and metastasis refer to the process in which tumor cells depart from the primary site and continue to grow in organs/tissues through various routes of transport, forming tumors of the same nature (metastatic tumors). The steps of tumor invasion and metastasis include: 1. Tumor cells degrade basement membrane and invade surrounding tissues; 2. Tumor cells invade blood or lymphatic vessels; 3. Tumor cells adhere to the vascular endothelium at the target site and grow to form the metastatic tumors; 4. The metastatic tumors invade to the surrounding area and spread throughout the body. The characteristics of tumor invasion and metastasis are heterogeneity, organ specificity, and dormancy phenomenon.

Upregulation of ciRS-7 can promote tumor cells invsion and metastasis in multiple cancers, including lung cancer 53, 54, HCC 11, CC 9, CRC 15, 55, BC 16, ESCC 17, 52, 61, PDAC 20, LSCC 21, and cholangiocarcinoma 62.

## ciRS-7 in various human cancers

Recently, several studies have revealed that abnormal ciRS-7 expression is correlated with various cancers, including lung cancer, HCC, CC, OS, melanoma, CRC, BC, ESCC, NPC, GC, PDAC, LSCC, and cholangiocarcinoma. The upregulation of ciRS-7 can serve as an oncogene to promote the progression of various tumors, especially in lung cancer, HCC, CRC, and ESCC. Furthermore, ciRS-7 overexpression has been correlated with a large tumor volume, advanced FIGO stage, deep infiltration, early metastasis, and poor survival. The correlation between clinicopathological parameters and ciRS-7 expression of different types of cancers are shown in **Table 1**. The manipulation of ciRS-7 (upregulation or downregulation) could influence the cell viability, growth, invasion, migration, apoptosis, and autophagy through miRNA-mRNA signaling in lung cancer, HCC, CRC, BC, and ESCC. The specific mechanisms and functional characterization of ciRS-7 in these cancers are presented in **Table 2** and **Table 3**.

### Lung cancer

Lung cancer is one of the most malignant tumors in world, with the second highest incidence rate and highest mortality rate in both males and females 1. The therapeutic effect and five-year survival rates of lung cancer are unsatisfactory, because most patients are diagnosed at an advanced stage 63. Therefore, it is necessary to explore early diagnostic and therapeutic strategies.

**Figure 1:**
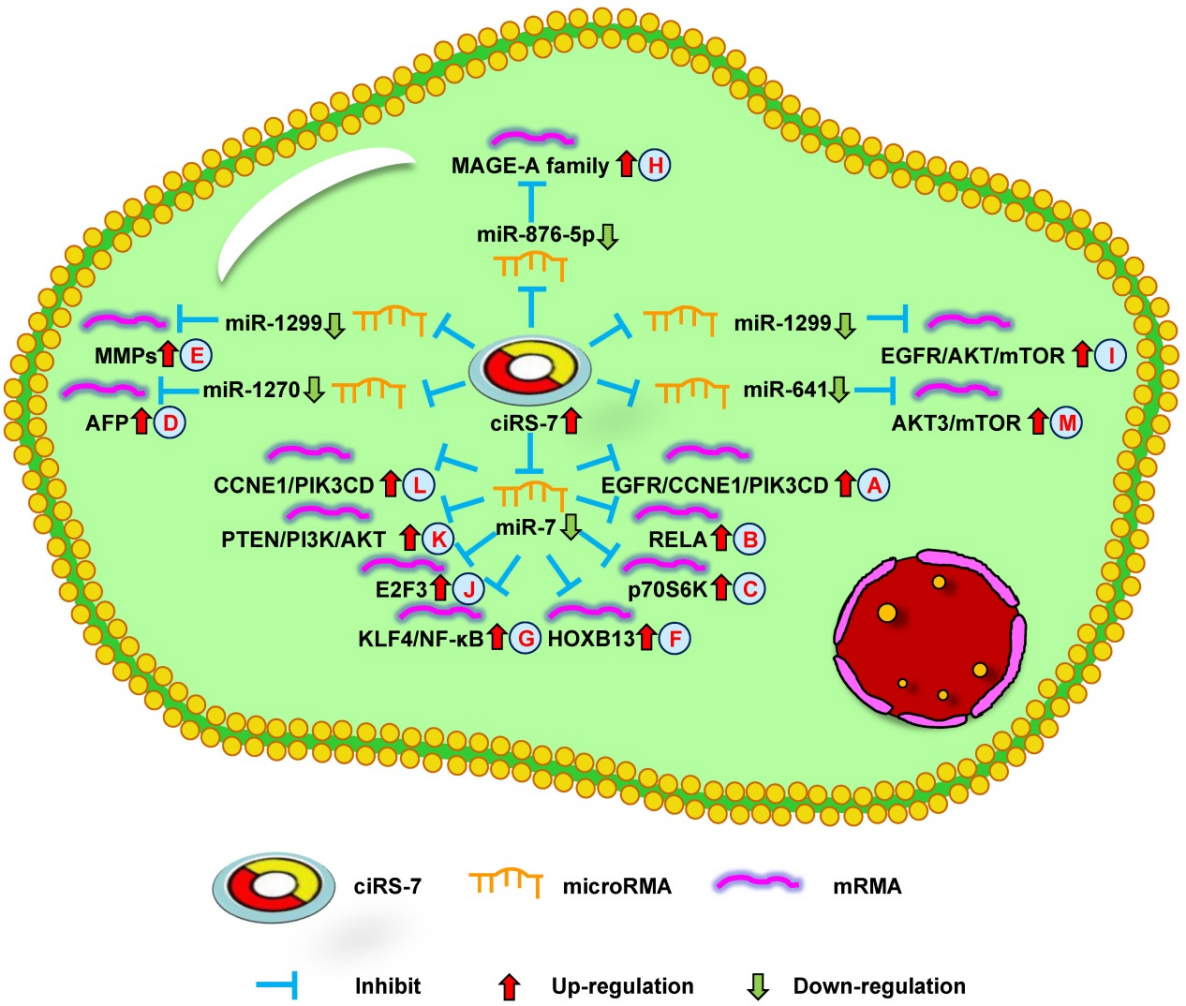
** Up-regulation of ciRS-7 mediates mechanisms involved in tumor progression.** (**A-C**). ciRS-7 could promote the expression of EGFR/CCNE1/PIK3CD (**A**), RELA (**B**), and p70S6K (**C**) by sponging miR-7. (**D**). ciRS-7 could upregulate AFP expression by sponging miR-1270. (**E**). ciRS-7 could function as a ceRNA of miR-1299 to enhance MMPs expression. (**F, G**). ciRS-7 could reactivate HOXB13 (**F**), and KLF4/NF-κB (**G**) signals by acting as a miR-7 sponge. (**H**). ciRS-7 increase MAGE-A family expression by functioning as a sponge of miR-876-5. (**I**). ciRS-7 targets EGFR-AKT-mTOR pathway by sponging miR-1299. (**J-L**), ciRS-7 upregulate E2F3 (**J**), PTEN/PI3K/AKT (**K**), and CCNE1/PIK3CD (**L**) expression through sponging miR-7.

**Figure 2:**
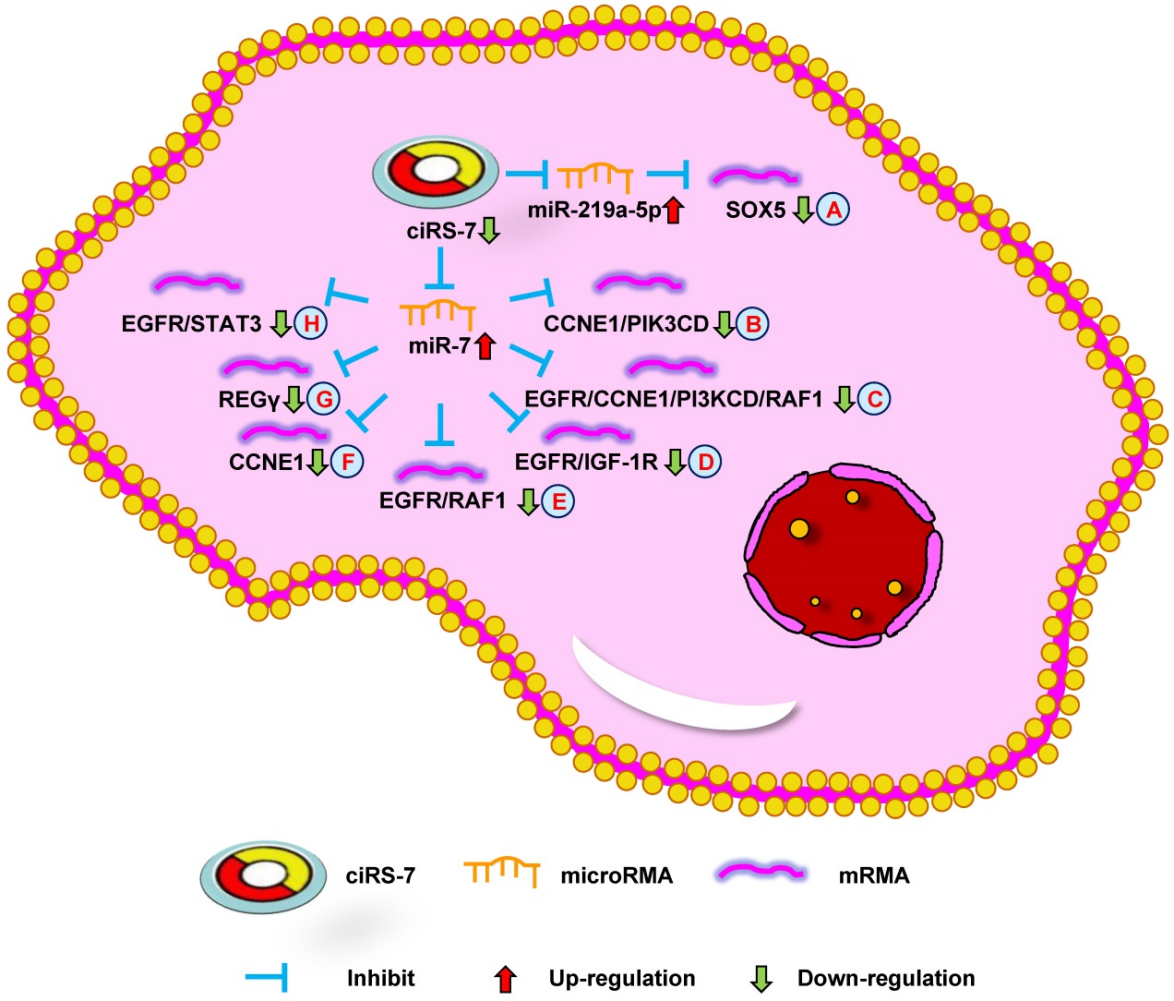
** Down-regulation of ciRS-7 mediates mechanisms involved in tumor progression.** (**A**). Knockdown of ciRS-7 could inhibit SOX5 expression by upregulating miR-219a-5p. (**B-H**). Knockdown of ciRS-7 suppress the expression of CCNE1/PIK3CD (**B**), EGFR/CCNE1/PI3KCD/RAF1 (**C**), EGFR/IGF-1R (**D**), EGFR/RAF1 (**E**), CCNE1 (**F**), REGγ (**G**), and EGFR/STAT3 (**H**) through targeting miR-7.

The study by Zhang et al. 10 indicated that ciRS-7 overexpression could promote non-small cell lung cancer (NSCLC) cell growth by sponging miR-7 to upregulate target genes, including EGFR, CCNE1, and PIK3CD (**Figure 1A**). Su et al. 53 reported that ciRS-7 increased NSCLC cell proliferation, invasion, migration, and apoptosis through the miR-7/RELA axis (**Figure 1B**). In addition, Li et al. 54 showed that a knockdown of ciRS-7 could inhibit NSCLC cell viability, migration, and invasion to inhibit SOX5 expression (**Figure 2A**). Moreover, Mao et al. 56 revealed that ciRS-7 contributed to PTX and CDDP chemoresistance through the EGFR/PI3K signaling pathway in lung adenocarcinoma. Yan et al. 64 found that a knockdown of ciRS-7 inhibited cellular proliferation and induced cell apoptosis in NSCLC. These findings show that ciRS-7 could serve as an oncogene to promote the progression of lung cancer.

### Hepatocellular carcinoma

HCC is another of the most common malignant tumors in the world 65. Due to the tendency for metastasis, the five-year survival rate of patients with HCC remains extremely low 66. Therefore, it is important to determine the biological mechanism of HCC and identify novel prognostic biomarkers and effective treatments to improve its prognosis.

The study by Yu et al. 11 reported ciRS-7 overexpression in HCC tissues compared with the adjacent non-tumor tissues. A knockdown of ciRS-7 inhibited the proliferation and invasion of HCC cells by targeting miR-7 to suppress CCNE1 and PIK3CD expression (**Figure 2B**). Xu et al. 67 showed that high ciRS-7 expression was correlated with microvascular invasion (MVI), younger age, and AFP level in HCC. ciRS-7 was also found to be an independent risk factor of MVI and to have a negative relationship with miR-7. The downregulation of miR-7 could increase the level of p70S6K mRNA and protein expression (**Figure 1C**). ciRS-7 may represent an HCC biomarker and novel therapeutic target for inhibiting hepatic MVI. ciRS-7 also promoted the proliferation and migration of HCC cells by sponging miR-1270 to upregulate AFP expression (**Figure 1D**) 51. Together, these studies support the conclusion that ciRS-7 could function as an oncogene to promote HCC progression.

### Cervical cancer

CC is a major cancer that threatens women's health. Despite the significant progress that has been made over the past few decades, the pathogenesis of CC remains poorly understood. Therefore, a sufficient understanding of CC tumorogenesis and progression may help us fight against it.

Zhou et al. 12 demonstrated that CC patients with high ciRS-7 expression had a large tumor volume, deep infiltration, advanced FIGO stage, early lymph node metastasis, and high incidence of HPV infection. The overexpression of ciRS-7 promoted the epithelial-mesenchymal transition (EMT) and growth of CC cells. Therefore, targeting ciRS-7 may represent a novel method of inhibiting CC progression. However, the underlying mechanism of ciRS-7 in CC remains unknown.

### Osteosarcoma

OS is a common primary malignant bone tumor, which is the leading cause of cancer-related death in both children and adolescents. Some patients obtained a poor prognosis or even developed other secondary malignant neoplasms 68. Thus, it is necessary to identify effective diagnostic markers and molecular mechanisms.

Researchers have shown that ciRS-7 was increased in OS cells and was negatively correlated with miR-7. Patients with high ciRS-7 expression had high Enneking stage, tumor size, and distant metastasis. The inhibition of ciRS-7 could lead to derepress levels of miR-7 and decrease OS cell migration. ciRS-7 could also promote EMT through the upregulation of N-cadherin and inhibition of E-cadherin. Xu et al. 13 demonstrated that ciRS-7/miR-7/EGFR/CCNE1/PI3KCD/RAF1 signaling could be used for the treatment of OS, functioning as a molecular target (**Figure 2C**). Together, these data demonstrate that ciRS-7 can function as an oncogene in OS progression.

### Melanoma

The estimated incidence of melanoma ranks fifth and sixth among males and females, respectively, in 2020 1. Metastatic melanoma has increased over the past few years, leading to an increased mortality rate 69. Therefore, there is an urgent need to identify effective diagnostic biomarkers and treatment for melanoma patients.

Zhang et al. 14 revealed the relationship between miRNA, proteins, and ciRS-7. Bioinformatics software was used to detect the correlation between ciRS-7 and miRNAs, and identified 15 candidates, including miR-7. They also predicted that 60 proteins could interact with ciRS-7, most of which participate in cancer-related biological processes. The bioinformatics software revealed that ciRS-7 could function as a ceRNA for the migration and invasion of melanoma. These findings suggest that ciRS-7 participates in melanoma progression and functions as a therapeutic target and prognostic biomarker.

### Colorectal cancer

The estimated incidence and mortality of CRC makes it the third leading cause of cancer-related death in both males and females 1. Although several studies have shown that some targets are associated with CRC, only a small number of targets are available for clinical treatment. Therefore, CRC pathogenesis is extremely important to explore therapeutic targets.

Both the studies by Tang et al. 15 and Weng et al. 55 reported high ciRS-7 expression in CRC tissues compared with the adjacent tissues and was significantly positively correlated with poor overall survival. The downregulation of ciRS-7 could inhibit cellular growth and invasion through the upregulation of miR-7 to inhibit EGFR/IGF-1R or EGFR/RAF1 expression in CRC cells, respectively (**Figure 2D and E**). Silencing ciRS-7 could suppress EGFR expression, which could be partially rescued with an miR-7 inhibitor. Moreover, ciRS-7 may play an important role in CRC progression. Tanaka et al. 70 showed that ciRS-7 overexpression could increase the level of PD-1 protein expression on the surface of CRC cells. ciRS-7 could result in a poor prognosis in an miR-7-independent manner in CRC. These results indicate that ciRS-7 can be used as a biomarker to diagnose CRC, and provide a novel method of CRC treatment.

### Breast cancer

BC is one of the most common cancers in females and represents a major threat to public health. One study showed that 30% of women are diagnosed with early-stage BC, which subsequently develops into metastatic BC due to limited treatment options 71. Therefore, molecular mechanisms are critical to improve the treatment of BC patients.

Sang et al. 16 revealed high ciRS-7 expression in triple negative breast cancer (TNBC) and the downregulation of ciRS-7 suppressed the invasion and migration of TNBC cells. ciRS-7 could function as a ceRNA of miR-1299 to increase cellular invasion and migration by enhancing MMP expression in TNBC cells (**Figure 1E**). The study by Yang et al. 57 demonstrated that ciRS-7 was increased while miR-7 was decreased in 5-FU-resistant BC cells. The inhibition of ciRS-7 increased the chemosensitivity of BC cells through upregulating miR-7 to suppress CCNE1 expression (**Figure 2F**). After a few months, it was found that ciRS-7 silencing decreased REGγ expression through relieving the competitive inhibition of miR-7 and strengthening the sensitivity of BC cells (**Figure 2G**) 58. Together, these studies imply that ciRS-7 inhibition plays an important role in increasing the sensitivity of chemotherapeutic drugs.

### Esophageal squamous cell carcinoma

Esophageal cancer is a common tumor of the gastrointestinal tract, which causes the death of patients annually throughout the world 72. However, the rate of early detection in patients is very low due to atypical clinical symptoms. There is an urgent need to clarify the progress of ESCC cells, which will help improve the diagnosis and treatment of this disease.

Li et al. 17 found that ciRS-7 expression was increased in ESCC and related to poor survival. In addition, ciRS-7 overexpression could induce the malignant development of ESCC, acting as an miR-7 sponge to reactivate HOXB13-NF-κB/p56 signaling (**Figure 1F**). Therefore, ciRS-7 may be a prognostic marker for ESCC. The study by Huang et al. 61 revealed similar results, showing that ciRS-7 could accelerate the invasion and migration of cells through miR-7-KLF4-NF-κB pathways in ESCC (**Figure 1G**). The inhibition of ciRS-7 might be a potential therapeutic target of ESCC treatment. Sang et al. 52 further reported that ciRS-7 could promote the progression of ESCC by functioning as a sponge of miR-876-5p to increase MAGE-A family expression (**Figure 1H**). Subsequently, the research team found that ciRS-7 sponged miR-1299 to inhibit ESCC cell autophagy by targeting the EGFR-AKT-mTOR pathway (**Figure 1I**) 73. Overall, these results demonstrate that ciRS-7 has oncogenic effects in ESCC and may represent a promising therapeutic target.

### Nasopharyngeal carcinoma

NPC is a malignant tumor of the head and neck. Although the incidence of NPC is not as high as that of other tumors, it deteriorates rapidly and is associated with a poor prognosis. Therefore, it is imperative to explore the pathogenesis and identify novel therapeutic targets of NPC.

Zhong et al. 18 demonstrated that ciRS-7 expression was increased in NPC and correlated with poor clinicopathological parameters. ciRS-7 accelerated the progression of NPC cells through sponging miR-7-5p to upregulate E2F3 expression both *in vitro* and *in vivo* (**Figure 1J**). Thus, ciRS-7 may be a potential target for the treatment of NPC patients. However, the potential mechanism of ciRS-7 in NPC remains to be explored.

### Gastric cancer

GC is a gastrointestinal malignant tumor and a main cause of cancer-associated death worldwide. Although GC can be cured by surgery in the early stages, many patients lose the opportunity for surgery at the time of the diagnosis 74. Therefore, the exploration of novel diagnostic biomarkers and treatment strategies will be of great help to us.

Li et al. 19 discovered that the downregulation of ciRS-7 decreased REGγ expression through sponging miR-7 to promote cellular apoptosis in GC. The study by Pan et al. 75 revealed that ciRS-7 was upregulated in GC and associated with poor survival. The overexpression of ciRS-7 promoted an aggressive behavior of GC cells by suppressing miR-7-involved PTEN/PI3K/AKT signaling (**Figure 1K**). In summary, ciRS-7 plays an important role in GC prognosis and may be used for the treatment of GC.

### Pancreatic adenocarcinoma

Pancreatic adenocarcinoma is one of the few malignant tumors with low morbidity but high mortality 1. Despite the great efforts made in the progress of PDAC, the five-year survival rate remains less than 5% 76. Therefore, it is necessary to explore novel diagnostic biomarkers and targeted therapies.

Liu et al. 20 found that the level of ciRS-7 expression was higher in PDAC tissues than in paracancer tissues. The expression of ciRS-7 was negatively correlated with miR-7. The authors found that silencing ciRS-7 inhibited cellular growth and invasion by upregulating miR-7 to suppress EGFR and STAT3 expression (**Figure 2H**). This data shows that ciRS-7 functions as a therapeutic target for PDAC.

### Laryngeal squamous cell carcinoma

Laryngeal carcinoma is the second most common malignant tumors of the respiratory system. Despite advances in treatment over the past decade, the clinical outcomes of patients with advanced LSCC have not obviously improved. Therefore, exploring physiological mechanisms and identifying new therapeutic targets represent the key breakthrough factors for LSCC treatment.

The study by Zhang et al. 21 discovered that high ciRS-7 expression was associated with poorly differentiated tumors, high tumor stage, poor prognosis, and lymph node metastases in LSCC. The overexpression of ciRS-7 increased the level of cellular migration, invasion, and growth in LSCC. ciRS-7 promoted LSCC progression through sponging miR-7 to upregulate CCNE1 and PIK3CD expression (**Figure 1L**). These findings suggest that ciRS-7 might offer a therapeutic option for inhibiting LSCC progression.

### Cholangiocarcinoma

CCC represents one of the most frequent malignant tumors of the liver after hepatocellular carcinoma, accounting for approximately 3% of gastrointestinal tumors 77. CCC is difficult to diagnose in its early stages, and surgical excision is the only possible treatment. Therefore, it is necessary to explore the molecular mechanism of CCC tumorigenesis and identify potential therapeutic methods.

Jiang et al. 22 revealed that ciRS-7 expression was higher in CCC tissues compared with that in normal tissues. The overexpression of ciRS-7 was found to be related to lymph node infiltration, advanced clinical stage, and neoplasm recurrence. Thus, ciRS-7 could potentially be used as an independent predictive factor for CCC. Moreover, Li et al. 62 found that ciRS-7 promoted the oncogenic behavior of CCC cells. ciRS-7 activated the AKT3/mTOR signaling pathway by combining with miR-641 in CCC cells (**Figure 1M**). In summary, the above data demonstrate that ciRS-7 plays a vital role in CCC development and functions as a potential therapeutic target for CCC.

## Conclusion and future perspectives

circRNAs have gained increased attention over the past decade. With the rapid progress of high throughput sequencing and bioinformatics technology, multiple circRNAs have recently been discovered. Although the function and modulation of most circRNAs remains unclear, studies have started to excavate the clinical significance and effect of various circRNAs. Recently, evidence supports the view that circRNA expression is related to tumor initiation, growth, and metastasis. In addition, circRNAs may play a vital role as a therapeutic target and prognostic factor for many human tumors 78.

Although progress has been made regarding the role of circRNAs, there are some questions that deserve attention. Firstly, although most circRNAs are expressed at relatively low levels in human tumors, more sensitive and precise techniques and equipment are required to investigate the function of circRNAs. Secondly, most of the current studies on circRNAs rely on the results of RNA-sequencing data. Therefore, these findings may introduce bias in clustering circRNAs for an authentic and predictive analysis. Thirdly, most circRNA sequences are similar to the mRNA produced by host genes. Hence, the verification of circRNAs, as well as the downregulation and overexpression, require careful design and detection. Finally, due to the extensive presence of circRNAs in living organisms, the current detection techniques may ignore circRNA subtypes or circRNAs with a low abundance which may have biological significance.

ciRS-7 plays a vital role in the onset and progression of various tumors, functioning as an oncogenic circRNA, which is upregulated in many human cancers (e.g., NSCLC, HCC, CC, OS, melanoma, CRC, BC, ESCC, NPC, GC, PDAC, LSCC, and CCC). Abnormal ciRS-7 expression in tumors is closely related to various clinicopathological characteristics (e.g., age, blood serum markers, tumor size, vascular invasion, TNM stage, lymph node metastasis, distant metastasis, overall survival, disease-free survival, and tumor recurrence). Extensive studies indicate that ciRS-7 expression is upregulated and can promote cell viability, growth, invasion, and migration, as well as inhibit apoptosis. Multiple mechanistic studies have demonstrated that ciRS-7 can function as a ceRNA of multiple miRNAs (miR-7, miR-219-5p, miR-1270, miR-1299, miR-876-5p, and miR-641) to inhibit the expression of downstream genes (e.g., Ki-67, EGFR, CCNE1, PIK3CD, NF-κB, SOX5, p70S6K, AFP, RAF1, IGF-1R, MAPK, MMPs, REGγ, HOXB13, p56, KLF4, MAGE-A family, AKT, mTOR, E2F3, PTEN, PI3K, AKT3, and STAT3). Although various biological processes have been identified, the involvement of ciRS-7 in tumor progression requires further exploration.

Importantly, recent studies indicate that ciRS-7 could be applied as a potential therapeutic target and a predictive factor in a wide range of tumors. At present, there are no preclinical trials that validate the use of ciRS-7 in tumor management. However, this may be a promising direction for further investigation. Although ciRS-7 has been verified to be up-regulated in a substantial number of human tumors, it is downregulated in some tumors, including both ovarian cancer 59, 79 and bladder cancer 80. Thus, the underlying mechanism remains unclear and requires further study.

Collectively, the recent studies of ciRS-7 in human tumors provide the groundwork for future therapeutic strategies. ciRS-7 has been identified to play a vital role as an oncogene and shows its function through ceRNA mechanisms. ciRS-7 is bound to make a huge contribution in fighting cancer.

## Figures and Tables

**Table 1:** Clinicopathological parameters of ciRS-7 in human tumors.

Tumor types	Clinicopathological features	References
Lung cancer	high TNM stage, more lymph nodes metastasis, shorted overall survival time	10
Lung cancer	advanced histopathological grade, larger tumour size, severer lymph node metastasis	53
Lung cancer	tumor size, lymph node metastasis, tumor node metastasis, shorter disease-free survival and overall survival time	64
Liver cancer	younger age, serum AFP, hepatic microvascular invasion	67
Liver cancer	tumor diameter, serum AFP, tumor satellite	51
Cervical cancer	large tumor size, advanced FIGO stage, deep invasion, metastatic lymph nodes, HPV infection	12
Osteosarcoma	high Enneking stage, tumor size, pulmonary metastasis	13
Colorectal cancer	tumor size, T stage, lymph node metastasis, poor overall survival	15
Colorectal cancer	advanced tumor stage, tumor depth and metastasis, poor patient survival, poor overall survival	55
Esophageal cancer	poor overall survival	17
Esophageal cancer	high pathological grade, lymph node metastasis, distant metastasis or recurrence, poor prognosis	52
Nasopharyngeal cancer	high clinical staging	18
Gastric cancer	poor overall survival	75
Pancreatic cancer	venous invasion, lymph node metastasis	20
Laryngeal cancer	high TNM stages, poorly differentiated tumours, lymph node metastasis, poor prognosis	21

**Table 2:** Functional characterization of ciRS-7 in various tumours.

Tumor types	Expression	Role	Function role	miRNAs	Related genes	References
Lung cancer	upregulation	oncogene	viability, growth, apoptosis	miR-7	EGFR, CCNE1, PIK3CD	10
Lung cancer	upregulation	oncogene	proliferation, invasion, migration, apoptosis	miR-7	RELA	53
Lung cancer	upregulation	oncogene	viability, migration, invasion, apoptosis	miR-219a	SOX5	54
Lung cancer	upregulation	/	proliferation, apoptosis	/	/	64
Liver cancer	upregulation	oncogene	proliferation, invasion	miR-7	CCNE1/PIK3CD	11
Liver cancer	upregulation	oncogene	proliferation, migration	miR-1270	AFP	51
Cervical cancer	upregulation	/	proliferation, apoptosis, invasion, migration	/	/	12
Osteosarcoma	upregulation	oncogene	vitality, apoptosis, growth, migration	miR-7	EGFR/CCNE1/PI3KCD/RAF1	13
Colorectal cancer	upregulation	oncogene	proliferation, invasion	miR-7	EGFR/IGF-1R	15
Colorectal cancer	upregulation	oncogene	proliferation, migration, invasion, apoptosis	miR-7	EGFR/RAF1	55
Breast cancer	upregulation	oncogene	migration, invasion	miR-1299	MMPs	16
Breast cancer	/	/	/	miR-7	CCNE1	57
Breast cancer	upregulation	oncogene	/	miR-7	REGγ	58
Esophageal cancer	upregulation	oncogene	proliferation, migration, invasion, metastasis	miR-7	HOXB13	17
Esophageal cancer	upregulation	oncogene	migration, invasion	miR-7	KLF4/NF-κB	61
Esophageal cancer	upregulation	oncogene	proliferation, migration, invasion	miR-876	MAGE-A family	52
Esophageal cancer	/	/	autophagy	miR-1299	EGFR/AKT/mTOR	73
Nasopharyngeal cancer	upregulation	oncogene	growth, glucose metabolism	miR-7	E2F3	18
Gastric cancer	upregulation	oncogene	growth, migration	miR-7	PTEN/PI3K/AKT	75
Pancreatic cancer	upregulation	oncogene	proliferation, invasion	miR-7	EGFR/STAT3	20
Laryngeal cancer	upregulation	oncogene	vitality, proliferation, migration, invasion	miR-7	CCNE1/PIK3CD	21
Gallbladder cancer	upregulation	oncogene	proliferation, migration, invasion	miR-641	AKT3/mTOR	62

**Table 3:** Main characteristics of the studies included in this review.

Study	Tumor types	Sample size (Normal : Tumor)	Detection Method	P value	TNM (p value)	LNM (p value)	DM (p value)	OS (p value)	DFS (p value)	Follow-up (months)	References
Zhang	Lung cancer	(60 : 60)	qRT-PCR	p<0.05	p=0.004	p=0.021	/	p=0.013	/	/	10
Su	Lung cancer	(128 : 128)	qRT-PCR	p<0.05	p=0.002	p=0.034	/	p<0.05	/	/	53
Li	Lung cancer	(30 : 30)	qRT-PCR	p<0.05	/	/	/	/	/	/	54
Yan	Lung cancer	(132 : 132)	qRT-PCR	p<0.05	p=0.024	p=0.003	/	p=0.001	p<0.001	/	64
Yu	Liver cancer	(35 : 35)	qRT-PCR	p<0.001	/	/	/	/	/	/	11
Xu	Liver cancer	(108 : 108)	qRT-PCR	p=0.13	/	/	/	/	/	/	67
Su	Liver cancer	(42 : 42)	qRT-PCR	p<0.001	/	/	/	/	/	/	51
Zhou	Cervical cancer	(352 : 352)	qRT-PCR	p<0.05	p=0.029	p=0.005	/	/	/	/	12
Xu	Osteosarcoma	(18 : 38)	qRT-PCR	p<0.001	/	/	/	/	/	/	13
Tang	Colorectal cancer	(40 : 40)	qRT-PCR	p<0.001	/	p=0.002	/	p=0.034	/	/	15
Weng	Colorectal cancer	(40 : 40)	qRT-PCR	p=0.0018	p=0.0002	p<0.0001	p=0.0162	p=0.0224	/	44	55
Yang	Breast cancer	(90 : 90)	qRT-PCR	p<0.05	/	/	/	/	/	/	58
Li	Esophageal cancer	(123 : 123)	qRT-PCR	p<0.05	p=0.048	/	/	p<0.05	p<0.05	/	17
Huang	Esophageal cancer	(29 : 29)	qRT-PCR	p<0.05	/	/	/	/	/	/	61
Sang	Esophageal cancer	(86 : 86)	qRT-PCR	p<0.01	/	/	/	/	/	12-60	52
Zhong	Nasopharyngeal	(20 : 44)	qRT-PCR	p<0.01	/	/	/	/	/	/	18
Pan	Gastric cancer	(102 : 102)	qRT-PCR	p=0.0023	/	p=0.0004	p=0.0205	p=0.0143	/	/	75
Liu	Pancreatic cancer	(41 : 41)	qRT-PCR	p=0.002	/	p=0.016	/	/	/	/	20
Zhang	Laryngeal cancer	(30 : 30)	qRT-PCR	p<0.001	p<0.05	p<0.001	/	p=0.02	/	/	21
Jiang	Gallbladder cancer	(54 : 54)	qRT-PCR	p<0.0005	p=0.044	p=0.006	/	p<0.0005	/	/	22
